# Supplementary material for: Identifying Quantitatively Differential Chromosomal Compartmentalization Changes and Their Biological Significance from Hi-C data using DARIC
Source: Res Sq. 2023 Apr 28:rs.3.rs-2814806. Preprint. [Version 1] doi: 10.21203/rs.3.rs-2814806/v1 (PMC10168473; doi:10.21203/rs.3.rs-2814806/v1)
Supplement: Supplement 1 [file NIHPPrs2814806v1-supplement-1.pdf]

Supplementary Files

This is a list of supplementary files associated with this preprint. Click to download.

- SupplementaryTable1.docx
- SupplementaryTable2new.xlsx
- supplementalfigures.pdf
